# Progress in the study of reproductive toxicity of platinum-based antitumor drugs and their means of prevention

**DOI:** 10.3389/fphar.2024.1327502

**Published:** 2024-02-13

**Authors:** Zhan Jin, Liu Zhao-Xia, Peng Fan-Ke, Zhang Wen-Juan, Wei Min-Li, Zeng Han-Yi

**Affiliations:** ^1^ Gannan Medical University, Ganzhou, China; ^2^ Department of Reproductive Medicine, First Affiliated Hospital of Gannan Medical University, Ganzhou, China; ^3^ Bazhong Central Hospital, Bazhong, China; ^4^ Department of Genetics at the School of Basic Medicine, Gannan Medical University, Ganzhou, China

**Keywords:** platinum, antitumor, reproductive toxicity, genotoxicity, embryonic developmental toxicity

## Abstract

Platinum-based antitumor drugs are broad-spectrum agents with unique mechanisms of action. Combination chemotherapy regimens based on platinum drugs are commonly used in cancer treatment. However, these drugs can cause various adverse reactions in the human body through different routes of administration, including reproductive toxicity, genetic toxicity, and embryonic developmental toxicity. Preventing adverse effects is crucial to enhance patients' quality of life and reduce healthcare costs. This article discusses the types and developmental history of antitumor active platinum compounds, their mechanisms of action, routes of administration, and their potential reproductive, genetic, and embryonic developmental toxicity. This text explores preventive measures based on animal experimental results. Its aim is to provide references for personalized treatment and occupational protection when using platinum drugs. The continuous progress of science and technology, along with the deepening of medical research, suggests that the application of platinum drugs will broaden. Therefore, the development of new platinum drugs will be an important direction for future research.

## 1 Introduction

In the 1960s, Rosenberg and others made the initial discovery that platinum (Pt) ions can suppress cell division by conducting an experiment on *Escherichia coli* using a direct current electric field (Demkowicz et al., 2016). Subsequently, their findings led to the exploration of a new area of research on the antitumour effect of Pt complexes by oncology researchers. Pt-based anticancer agents have found broad application in various solid tumours like lung cancer, ovarian cancer, and head and neck tumours (Rossi and Di Maio, 2016; Kitamura et al., 2020; Zhang et al., 2022). Antitumor active platinum compounds are compounds comprising of platinum metal ions, which interact with deoxyribonucleic acid (DNA) through a mechanism that creates several structural adducts. This process triggers cellular responses leading to apoptosis in tumour cells (Galluzzi et al., 2012). However, it also potentially damages normal cells (Oun et al., 2018; Ben Ayed et al., 2020) and impacts healthcare workers exposed to them for a prolonged duration via skin and respiratory tract (Lehmann and Williams, 2020). Cisplatin is one of the treatments known for its adverse effects on systemic organs such as the uterus and ovaries (Albayrak et al., 2021; Bhardwaj et al., 2023), leading to ovarian endocrine disruption, follicular growth and developmental disorders, as well as liver and kidney function damage (Pabla and Dong, 2008; Gong et al., 2021). Consequently, it is crucial to investigate the mechanism behind cisplatin’s toxic effects and develop strategies to protect occupational health against antitumor active platinum compounds. The paper outlines various types, structures, mechanisms of action, routes of administration, potential reproductive and genotoxic effects, and embryonic developmental toxicity associated with antitumor active platinum compounds. Preventive measures are also discussed with the aim of exploring new avenues for personalised treatment, shielding pre-chemotherapy fertility and aiding medical staff preservation.

## 2 Types of platinum drugs and their chemical structures

### 2.1 Cisplatin

Currently, there are three primary types of platinum-based anticancer drugs in clinical use in China: cisplatin, carboplatin, and oxaliplatin (Kelland, 2007). Originally, platinum was employed as an electrode material, and its electrolysis by-products were discovered to be effective in halting normal *E. coli* division (Rosenberg et al., 1965). As a result of this groundbreaking finding, the first generation of platinum-based anticancer drug—cisplatin, was formulated utilising antitumor active platinum compounds (Osieka and Schmidt, 1979). Cisplatin (Table 1), also referred to as cis-dichlorodiammineplatinum, is an inorganic metal complex that has a simple planar tetragonal chemical structure, composed of a divalent platinum ion situated at the centre, two amino ligands and two chlorido ligands functioning as the reactive groups (Zhou et al., 2020). And it exhibits significant therapeutic benefits for a range of malignant tumours, including breast cancer, ovarian cancer, colorectal cancer, and others. However, long-term administration of cisplatin, a non-specific chemotherapeutic medication, poses a risk of nephrotoxicity to normal tissues and cells in the body (Qi et al., 2019). Adverse effects include acute renal impairment, hypomagnesemia, and hypertension amongst others, with acute kidney injury being the most prevalent and severe manifestation (Zhang J. et al., 2021). Furthermore, tumour cells' development of resistance to cisplatin as a chemotherapeutic agent due to attenuating apoptosis resulting from DNA damage is a constraint (Siddik, 2003).

**TABLE 1:** Chemical structures of platinum drugs.

Generation	Platinum drugs	Chemical structures	Listed timing (s)	Listed of country
I	Cisplatin (Zhou et al., 2020)	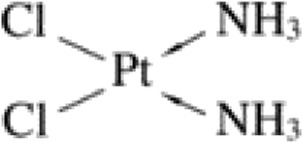	1978	Japan/Italy
II	Carboplatin (Zhou et al., 2020)	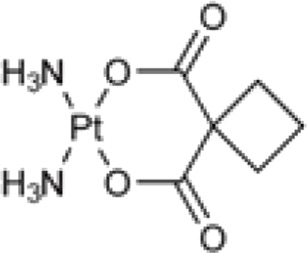	1986	America
II	Nedaplatin (Zhou et al., 2020)	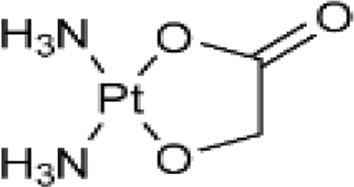	1995	Japan
III	Oxaliplatin (Zhou et al., 2020)	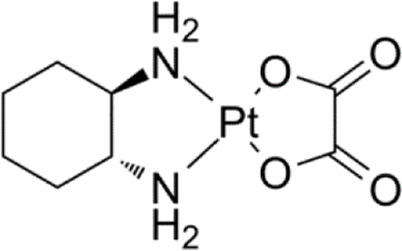	1996	France
III	Satraplatin (Choy, 2006)	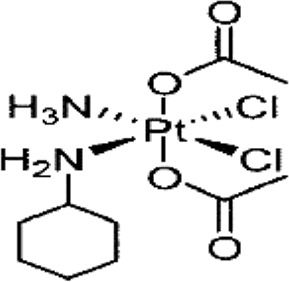	1999	America
III	Lobaplatin (Zhou et al., 2020)	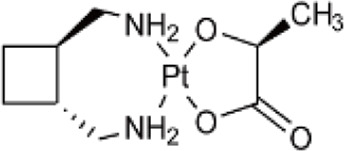	2005	China

### 2.2 Carboplatin

Carboplatin (Table 1), a second generation platinum-based chemotherapy drug, has been developed as a result of cisplatin, And it is also known as cis-diamminediyl (1,1-cyclobutanedioic acid) platinum, displaces the two chlorido ligands present in cisplatin with cyclobutanedicarboxylic acid (Zhou et al., 2020). Consequently, carboplatin does not attach to a wide range of plasma proteins when compared to cisplatin (Dilruba and Kalayda, 2016; Hu et al., 2017), with a high degree of biosafety and significantly reduced toxicities (Allen et al., 2020), primarily thrombocytopenia caused by myelosuppression (Ruggiero et al., 2013), as well as less common occurrences of nephrotoxicity, neurotoxicity, ototoxicity, and dosage-limiting nausea and vomiting. However, carboplatin exhibits a comparable anticancer profile to cisplatin and has been shown to display cross-resistance to cisplatin in various cancer types (Wagstaff et al., 1989).

### 2.3 Nedaplatin

Nedaplatin (Table 1) is a second-generation platinum anticancer medication formed through the substitution of two chlorido ligands in cis-platinum with ethanoic acid, having the full name cis-ethanolic acid diammine platinum (Zhou et al., 2020). This drug is highly soluble in water and mitigates nephrotoxicity by modifying the drug distribution within the kidney. The dose-limiting toxicity of nedaplatin is comparable to that of carboplatin, causing thrombocytopenia due to myelosuppression. However, its nephrotoxic and gastrointestinal side effects are lesser than those induced by cisplatin. Therefore, nedaplatin has proven more beneficial than cisplatin for patients exhibiting resistance to the latter (Shimada et al., 2013).

### 2.4 Oxaliplatin

To prevent cross-resistance to cisplatin and carboplatin combination therapy, the third generation platinum anticancer drug, oxaliplatin, was developed. Oxaliplatin (Table 1) is also known as [oxalate (2-)-O, O′][1R,2R-cyclohexanediamine-N, N’] platinum-(II). Oxaliplatin belongs to the third generation of platinum antitumour drugs. Its center is platinum, with a 1,2-diaminocyclohexane group that replaces the amino ligands of cisplatin (Zhou et al., 2020), and the oxalate coordinated to platinum acts as an active living group. Oxaliplatin has shown substantial therapeutic effects *in vitro* and *in vivo* on several tumour cell lines, including those that are susceptible to resistance to cisplatin and carboplatin (Raymond et al., 1998).

### 2.5 Lobaplatin

Lobaplatin (Table 1) is composed of an approximate 50/50 mixture of two diastereoisomers with R,R,S- and S,S,S-configurations, with the complete name 1,2-diaminomethyl-cyclobutane-lactate platinum. And it is categorized as a third-generation platinum-based antitumour medication (Zhou et al., 2020). Furthermore, Lobaplatin affects gene expression levels, including c-mye, besides its effect on DNA synthesis and replication. Lobaplatin does not cause cross-resistance to cisplatin and has shown effectiveness in overcoming cross-resistance between cisplatin and carboplatin in certain clinical tumours. However, it can lead to thrombocytopenia as a side-effect due to decreased activity of haematopoietic precursors in the bone marrow. Lobaplatin is primarily used in the clinical management of breast cancer, chronic myeloid leukaemia, and non-small cell lung cancer (Mckeage, 2001).

### 2.6 Satraplatin

The chemical name of satraplatin (Table 1) is bis-(acetato)-ammine dichloro-(cyclohexylamine)platinum (IV). Currently marketed platinum drugs, with the exception of satraplatin, must be administered intravenously. However, satraplatin was the first platinum drug to be administered orally in clinical studies, which means it can be conveniently used clinically through intermittent dosing (Bhargava and Vaishampayan, 2009). Satraplatin and its major metabolite, JM-118, have exhibited antitumour activity *in vitro*, *in vivo*, and in clinical trials (Choy et al., 2008). Furthermore, due to its greater hydrophobicity than cisplatin, satraplatin has demonstrated significant efficacy in cisplatin-resistant tumour cell lines. It also displays activity against lung, ovarian, and prostate cancers, and research has shown that a combination with radiotherapy in the treatment of lung and head and neck cancers can achieve superior therapeutic outcomes (Choy, 2006). Table 1 illustrates the chemical structures of platinum drugs.

## 3 Mechanism of action of platinum-based drugs

**FIGURE 1:**
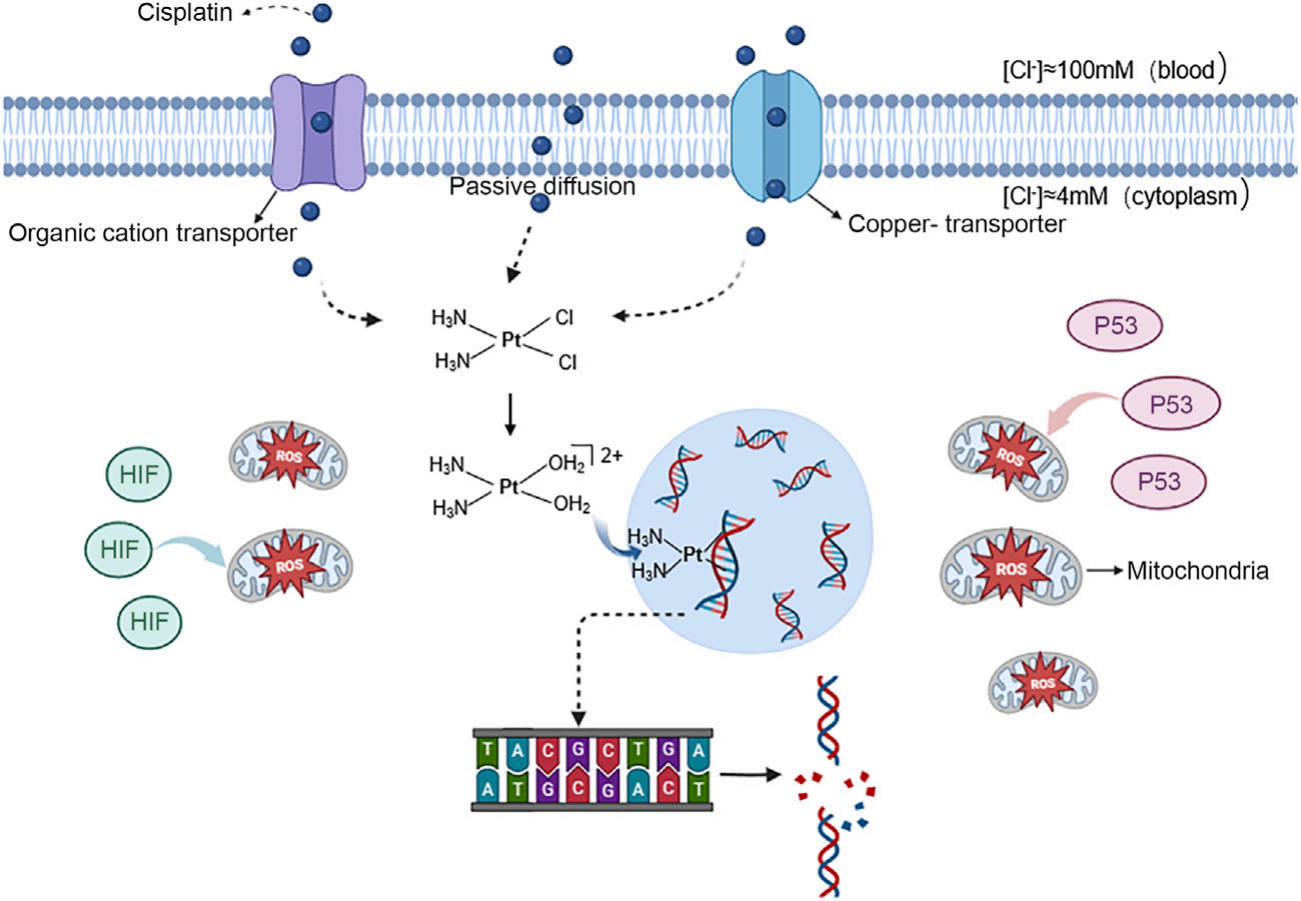
Mechanism of action of cisplatin in entering cancer cells and causing apoptosis antitumor active platinum compounds are represented by the blue spheres. These ions can enter the nucleus of tumour cells through free diffusion and transport by the organic cation transporter proteins OCT1-3 and copper transporter protein 1. Once inside,they bind to DNA, breaking the hydrogen bond between the purine group and cytosine on the two polynucleotide strands, causing DNA damage. And the expression of P53 and HIF increases and directly targets mitochondria, causing them to produce excessive ROS and induce the onset of apoptosis.

The mechanism of action for platinum antitumour agents can be divided into four stages: transmembrane transport, hydration-dissociation, target migration, and binding to DNA (Zhang et al., 2022). This blocks DNA replication, leading to inhibited cell division and proliferation, and ultimately apoptosis. Firstly, antitumor active platinum compounds enter tumour cells through passive diffusion and transport proteins, including organic cation transporter OCT1-3 (organic cation transporter 1–3) and copper-transporter 1 (CTR1). Afterward, these antitumor active platinum compounds transform into cationic hydrates in the cytoplasm and ultimately enter the nucleus of the tumour cell to form intra- and interstrand crosslinks with DNA (Johnstone et al., 2016). The most nucleophilic DNA site is the N7 position of guanine, which is exposed in the major groove and is preferentially platinated. This disrupts hydrogen bonding between the purine group and cytosine on the two polynucleotide strands, causing abnormal DNA double helix structure and inflicting DNA damage (Dasari and Bernard Tchounwou, 2014). The resultant effect may cause cell cycle arrest and DNA damage in rapidly proliferating tumour cells. In rapidly proliferating cancer cells, this damage can impede cell cycle progression and trigger apoptosis (Raudenska et al., 2019). Simultaneously, the augmented expression of P53, hypoxia-inducible factor (HIF) and other cytoplasmic proteins are directly conveying to the mitochondria. This results in the production of excessive reactive oxygen species (ROS) that initiate the onset of apoptosis (Refer to Figure 1) (Zhou et al., 2020; Zhang et al., 2022). Cisplatin, carboplatin, oxaliplatin and nedaplatin share a common chemical structure comprising of platinum, carrier groups and leaving groups, and undergo a similar mechanism of cytotoxicity (Zhang et al., 2022). However, only cisplatin is known to induce nephrotoxicity. Recent research suggests that organic cation transporters play a crucial role in the nephrotoxicity of platinum agents. Specifically, OCT2 mediates the transport of cisplatin and is the main cause of cisplatin-induced nephrotoxicity. Additionally, MATE1 protects against cisplatin-induced nephrotoxicity. The luminal efflux transporter, MATE2-K, and OCT2 did not transport carboplatin and nedaplatin, while oxaliplatin, a superior substrate Carboplatin and nedaplatin were not transported by these transporters (Yonezawa, 2012).

Furthermore, researchers have attained more efficient molecularly targeted therapy by chemically altering first-line platinum drugs (Dhar et al., 2011), in addition to the traditional mechanism of action of such drugs. For instance, utilizing Trastuzumab as a carrier to encapsulate and transport platinum-based drugs to target cancer cells that overexpress HER2 can enhance the toxicity of platinum-based drugs to their target cells and significantly reduce their side effects on humans (Huang et al., 2015). However, it is important to note that molecular targeted therapies may result in the loss of normal tissue functions due to the expression of cancer cell envelope proteins in some normal tissue cells. For instance, Luteinizing Hormone-Releasing Hormone (LHRH) and Human Epidermal Growth Factor Receptor 2 (HER2) have been used as receptor-modified platinum precursor drugs for the treatment of breast cancer by some scholars (Huang et al., 2015; Li et al., 2015). The platinum drugs modified by the package can also bind more precisely to normal tissue cells expressing the above two receptors. Therefore, it is necessary to determine whether this method will also result in reproductive toxicity of platinum-based drugs.

Figure 1丨antitumor active platinum compounds are represented by the blue spheres. These ions can enter the nucleus of tumour cells through free diffusion and transport by the organic cation transporter proteins OCT1-3 and copper transporter protein 1. Once inside, they bind to DNA, breaking the hydrogen bond between the purine group and cytosine on the two polynucleotide strands, causing DNA damage. And the expression of P53 and HIF increases and directly targets mitochondria, causing them to produce excessive ROS and induce the onset of apoptosis.

## 4 Routes of administration of platinum-based drugs

Platinum drugs are usually classified as injectable solutions and oral tablets (Choy et al., 2008). However, Satraplatin, a platinum-based drug administered via inhalation, has not received approval from the FAD (Bhargava and Vaishampayan, 2009). The primary clinical methods of administration for platinum-based drugs are 1) intravenous injection, 2) intraperitoneal instillation, and 3) catheterised arterial instillation. Intravenously administered platinum drugs include cisplatin (Crom et al., 1984), carboplatin (Van Der Vijgh, 1991), nedaplatin (Sasaki et al., 1991), oxaliplatin (Brienza et al., 1999), and lobaplatin (Welink et al., 1999). Saline-based solutions are generally used for cisplatin and nedaplatin, while glucose-based solutions are used for carboplatin and oxaliplatin (Yoshioka et al., 1999; Alassadi et al., 2022).

Cisplatin is occasionally administered through intraperitoneal instillation. For instance, in the clinical management of advanced ovarian cancer, a perfusion solution that contains cisplatin will be circulated cyclically and precisely using a thermal therapeutic instrument. It is then infused into the abdominal cavity and maintained for a specified time, with the objective of preventing and treating malignant tumor implantation and metastasis (Zivanovic et al., 2015). In certain instances of interventional chemotherapy combined with drugs, catheter arterial infusion chemotherapy therapy is typically employed, for example, cisplatin combined with 5-fluorouracil (5-FU) intra-arterial infusion chemotherapy which has been shown to effectively treat locally advanced pancreatic cancer (Sasada et al., 2008).

Meanwhile, during intravenous drug administration for cancer patients, platinum-based anticancer drugs may be released into the air through vaporisation, leading to indirect exposure or inhalation of platinum agents through the skin or respiratory routes (Hori et al., 2020). Research by the International Agency for Research on Cancer (IARC) has classified cisplatin and other platinum-based anticancer drugs commonly found in hospital air as class 2A carcinogens (Hori et al., 2020). Thus, platinum-based drugs are unintentionally impeding human germ cells and impacting the health of patients and healthcare workers' children, while they are being given through injection to treat cancer (Abdel-Latif et al., 2022).

## 5 Reproductive toxicity and genotoxicity of platinum drugs

Platinum drugs, which are frequently employed chemotherapeutic agents, are extensively utilised in treating a range of malignancies, including ovarian, bladder, lung and liver cancer, among others. Nonetheless, these drugs not only eliminate cancerous cells, but also damage healthy cells, unavoidably leading to side effects. Studies indicate that male rats injected with cisplatin experienced significant reproductive system damage compared to the control group (Sawhney et al., 2005). Biochemical analysis revealed decreased activities of superoxide dismutase (SOD), catalase (CAT), glutathione peroxidase (GSH-Px) and reduced glutathione (GSH), while the TBARS level increased significantly. At the protein level, a significant decrease in TBARS activity was detected. Additionally, the expression of steroid acute regulatory protein (StAR) decreased, limiting the biosynthesis of steroid hormones. This resulted in a decrease in serum testosterone levels at the hormone level. A decrease in the weight of the testes, pituitary gland, and seminal vesicles was observed, along with a significant decrease in the number of mature spermatozoa and spermatozoon viability in the epididymis, and an increase in abnormal spermatozoa, at both the organ weight and sperm parameters level. Additionally, a decrease in average spermatogenesis was observed at the tissue morphology level. A decrease in the mean diameter of seminiferous tubules, as well as thinning of the seminiferous epithelium were observed along with vacuolisation of spermatogonia, significant maturation disorders and perivascular fibrosis (Huang et al., 1990; Ilbey et al., 2009; Ciftci et al., 2011; Abdel-Latif et al., 2022). The study on cisplatin-induced reproductive toxicity in female mice found that histopathology indicated a reduction in the count of primary and secondary follicles, disappearance of Grave’s follicles, congestion of the interstitial space, and significant infiltration of monocytes and neutrophils. A noteworthy rise in corpus luteum in the medulla was also observed in the ovary. Endothelial epithelial debridement and degeneration were observed in the uterus, accompanied by a reduced number of endothelial glands, congestion in the lamina propria, and considerable mesenchymal infiltration. Furthermore, an Immunohistochemical analysis discovered elevated apoptosis in the uterine mucosa and uterine glands of female rats that received cisplatin injections. A reduction in anti-Müllerian hormone (AMH), oestrogen (E2), and reduced glutathione activity, accompanied by an elevation in reactive oxygen species (ROS) like superoxide anion, hydrogen peroxide, and hydroxyl radicals, contribute to changes in the number of free radicals in the uterus. These changes are responsible for the endothelial function alteration of the uterus. An elevated number of free radicals can increase lipid peroxidation by disrupting the fluidity and permeability of the biofilm. This leads to oxidative stress and can cause damage to the ovaries and uterus. Additionally, an increase in pro-inflammatory cytokines, including nuclear factor (NF-κB), tumour necrosis factor-α (TNF-α), interleukin-1 (IL-1β), and interleukin-6 (IL-6) levels, as well as cyclooxygenase-2 (COX-2) and nitric oxide synthase (iNOS), has been observed (Kaygusuzoglu et al., 2018; Said et al., 2019; Ayres et al., 2020; Huang et al., 2021).

Platinum drugs not only induce oxidative stress and reproductive toxicity in rat tissues, but they also cause genotoxicity. This is supported by the presence of chromosomal aberrations and micronucleus formation in bone marrow cells, as stated in reference (Zhang et al., 2020). A study aimed to establish the genotoxicity of cisplatin and carboplatin on cultured human lymphocytes unveiled that these two drugs significantly raised the probability of chromosomal aberrations (CA) and sister chromatid exchanges (SCEs) in lymphocytes as compared to control cases. Both drugs had a similar CA inducing capability and affected the mitotic index of the cells (Azab et al., 2019). An escalation in the incidence of micronuclei in the peripheral blood cells of mice that underwent continuous injection of cisplatin was detected during the assay of micronuclei (Prasad and Prasad, 2021).

## 6 Effects of platinum on early embryo development

Antitumor active platinum compounds have a deleterious effect on early embryonic development, in addition to being reproductive and genotoxic in mice. Studies have demonstrated that administering platinum compounds continuously to pregnant female rodents resulted in a considerable reduction in maternal body weight gain. Exposure to platinum compounds during the formation of tissue and organs in the foetus led to a noteworthy decrease in embryo survival, and an increase in embryo mortality and resorption. In addition to lethality, platinum drugs result in growth retardation and developmental anomalies in surviving foetuses, as demonstrated by the dose-dependent decrease in body weight and the presence of external, visceral, and skeletal malformations. External malformations present were umbilical hepatic prominence and an obvious cleft palate. Visceral anomalies comprised dilated nostrils, anophthalmia or microphthalmia, concomitant cardiomegaly, pulmonary hypoplasia, hepatomegaly, and renal pelvic dilatation. The skeletal anomalies observed in affected individuals included abnormally large fontanelles, delayed cranial ossification, and insufficiently ossified parietal and thoracic bones. Additionally, there were reduced or absent numbers of phalanges, sacral vertebrae, and caudal vertebrae. Furthermore, rib protrusions overgrew, forming extra ribs (Kai et al., 1989; Ognio et al., 2003; Hassan et al., 2016). Furthermore, Osterauer R et al. discovered in their research examining the impact of platinum absorption on early embryos in zebrafish and Ramsay’s horn snail that elevated concentrations of antitumor active platinum compounds resulted in a slowed rate of embryo hatching in both species (Osterauer et al., 2009). The research findings indicate that elevated levels of platinum lead to impaired early embryonic development accompanied by fetal death and deformities. This implies that platinum therapy for cancer treatment may impede early human embryonic growth.

## 7 Preventive measures to reduce reproductive and genotoxicity of platinum agents

Platinum-based anti-cancer drugs are commonly used in clinics, however, they carry the risk of inducing reproductive and genotoxicity which can cause permanent harm to the body. As a result, numerous researchers have dedicated themselves to investigating the mechanism of platinum drugs to prevent or lessen their toxic effects.Studies indicate that numerous chemical drugs and herbs possess the capability to hinder oxidative stress caused by cisplatin by eradicating free radicals and increasing the activity of antioxidant enzymes, ultimately providing reproductive and genotoxic protection against cisplatin. Studies demonstrate that sodium selenate, ellagic acid, and rutin can function as antioxidants to augment the performance of glutathione peroxidase 4 (GPX4) and prevent iron-induced cell death caused by elevated levels of reactive oxygen species resulting from platinum exposure. Consequently, this reduces severe pathological histomorphological modifications induced by platinum on the foetus and placenta as well as minimising the likelihood of degeneration and apoptotic cell death of spermatogonia in the testes (Türk et al., 2008; Hassan et al., 2016; Aksu et al., 2017). Research has demonstrated that the administration of cisplatin injection to pregnant rats resulted in a higher percentage of foetal mortality. Additionally, there was a decrease in the average number of foetuses, placental weight, and an increase in dwarf foetuses and subcutaneous haemorrhages. The placenta underwent severe pathological changes due to the production of free radicals and oxidative stress. Histopathological examination revealed trophoblastic necrosis, thickening, and hyalinisation of megaloblasts. However, after the administration of sodium selenate, it was found to significantly attenuate the adverse effects of cisplatin on the foetus and its placenta. This suggests that sodium selenate has a protective effect against the teratogenic effects of cisplatin. Male rats injected with cisplatin and then given ellagic acid by tube feeding showed significant improvement in the reduction of testis, epididymis, seminal vesicle, and prostate weights. Additionally, ellagic acid ameliorated the decrease of epididymal sperm concentration and viability, and reduced the cisplatin-induced sperm abnormality rate. Furthermore, the activities of glutathione peroxidase (GSH-Px) and catalase (CAT) were increased. Additionally, the degeneration, necrosis, interstitial oedema, and reduction in the thickness of the germinal cell layer of the seminiferous tubules were reversed. In this study, rats were injected with cisplatin and given rutin. The researchers evaluated spermatological parameters in the testis and found an increase in the density of spermatozoa in the cauda epididymis, as well as a decrease in the percentage of dead spermatozoa. Furthermore, it has been reported that male rats injected with cisplatin and given Liouweidihuangwan or Sanqi orally experienced a significant improvement in sperm counts and viability, resulting in a reduction in abnormal spermatozoa and micronuclei rates. Compared to mice injected with cisplatin, the treatment resulted in reduced serum malondialdehyde (MDA) levels and increased levels of total superoxide dismutase (T-SOD), glutathione peroxidase (GSH-Px), and catalase (CAT). This led to a significant inhibition of cisplatin-induced oxidative stress (Zhang et al., 2020; Zhang K. et al., 2021). Furthermore, research has demonstrated that astragalus polysaccharide functions as a chemo-sensitivity enhancer, potentially increasing the sensitivity of SKOV3 cells to cisplatin by activating the JNK pathway. This suggests that it may have utility in the treatment of ovarian cancer (Li et al., 2018)^.^

In a separate investigation, the inclusion of vitamin C was found to not only hinder the rise of reactive oxygen species count and decrease the likelihood of DNA damage and apoptosis, but also successfully prevent carboplatin from reducing the mouse oocytes' sperm binding ability and potential for fertilisation by interfering with their meiotic mechanism (Ahmed et al., 2011; Zhou et al., 2019). There is data to suggest that metformin can protect against cisplatin-induced apoptosis and genotoxicity in rat bone marrow cells. Metformin reduces the percentage of apoptotic cells and reactive oxygen species levels within bone marrow cells exposed to cisplatin in rats. Furthermore, it increases the level of erythrocytes and leukocytes within peripheral rat blood. This makes metformin a potentially effective adjuvant to cisplatin in the treatment of cancer (Cheki et al., 2021). However, further investigation is required to determine whether these drugs that prevent or reduce the reproductive toxicity and genotoxicity of platinum agents have an impact on the anticancer effects of the agents themselves.

## 8 Summary and outlook

This paper discusses the chemical types and structures of platinum-based antitumour drugs, their mechanisms of action, routes of administration, and potential reproductive, genotoxic, and embryotoxic effects. The paper also explores means of preventing these effects, providing valuable insights for the research and development of platinum-based antitumour drugs. Additionally, it serves as a reference for addressing potential clinical injuries associated with platinum drugs.

Platinum-based drugs are widely used as anti-malignant drugs in clinical applications. Their main mechanism of action is to form cross-links with DNA, inhibiting DNA replication and transcription, leading to apoptosis and necrosis. However, this mechanism also causes damage to normal cells while killing tumour cells. Numerous animal experiments have confirmed that platinum drugs can cause damage to the reproductive system, chromosomal aberrations, abnormal embryonic development, and other issues. Furthermore, studies have indicated that medical personnel who are exposed to platinum drugs for extended periods may come into contact with platinum agents indirectly through the skin and respiratory routes, suggesting a certain degree of occupational health risk. It has been demonstrated that certain chemical drugs and Chinese herbal medicines can reduce oxidative stress caused by platinum drugs by eliminating free radicals and enhancing the activity of antioxidant enzymes. This can play a protective role against the reproductive toxicity and genotoxicity caused by platinum drugs. This provides an effective way to explore methods of mitigating the toxicity and side effects of platinum drugs.

In conclusion, while platinum drugs present a significant advantage in antitumour therapy, there are still limitations, such as drug resistance and toxic side effects. Therefore, the focus should be on designing an ideal combination of platinum drugs that aligns with the mechanism of action of synergistic therapy, adjusting pharmacokinetic parameters, and improving the local concentration of drugs, aiming to emphasise the effect of targeted therapy. Undoubtedly, this is a promising direction for future development. Moreover, clinical studies have demonstrated considerable variability in the toxicity induced by chemotherapy across patients, despite consistent administration of the identical chemotherapy plan and dosage. Hence, the establishment of a collaborative, multi-centre trial to investigate the effect of genetic polymorphisms on the response to platinum-based chemotherapy is advocated. This will endeavour to explore the molecular basis for inter-individual contrasts in platinum drug toxicity and ultimately lead to personalized treatment with platinum-based drugs. Furthermore, it is imperative for the management to develop scientifically grounded and rational policies, as well as specific protective measures in order to address issues involving occupational exposure. These policies should aim to minimise health risks for those exposed, whilst simultaneously improving the safety of clinical drug use.
